# Multiyear Soil–Fruit Transfer Dynamics of Macro- and Trace Elements in Raspberry (*Rubus idaeus* L.) Under Field Conditions

**DOI:** 10.3390/plants15071107

**Published:** 2026-04-03

**Authors:** Ionela Ramona Zgavarogea, Nadia Paun, Claudia Sandru, Violeta-Carolina Niculescu, Ana Maria Nasture, Augustina Mirabela Pruteanu, Irina-Aura Istrate, Oana-Romina Botoran

**Affiliations:** 1 National Research and Development Institute for Cryogenic and Isotopic Technologies—ICSI Ramnicu Vâlcea, 4th Uzinei Street, P.O. Box Raureni 7, 240050 Ramnicu Valcea, Romania; ramona.zgavarogea@icsi.ro (I.R.Z.); ana.nasture@icsi.ro (A.M.N.);; 2 National Institute of Research—Development for Machines and Installations Designed for Agriculture and Food Industry—INMA, 013813 Bucharest, Romania; 3 Faculty of Biotechnical Systems Engineering, National University of Science and Technology Politehnica Bucharest, Splaiul Independentei 313, 060042 Bucharest, Romania

**Keywords:** raspberry (*Rubus idaeus* L.), soil–plant transfer, transfer factors, macro- and trace elements, lithium and strontium

## Abstract

Understanding the soil–plant transfer of both essential and non-essential elements is crucial for evaluating the crop nutritional quality, environmental interactions, and food safety. This study delivered a multiyear and multielement assessment under field conditions of the element uptake, translocation, and accumulation in raspberry (*Rubus idaeus* L.), based on data collected over two growing seasons (2024–2025) in two contrasting Romanian agroecosystems. Two commercial cultivars (Opal and Delniwa) were investigated under fertilized and unfertilized conditions. The concentrations of essential macroelements such as Ca, Mg, Na, and K, as well as trace elements (Li and Sr), were determined in soils and fruits using ICP-OES and AAS. The soil–fruit transfer was quantified through the transfer factor, assisted by a robust statistical framework which integrated spatial–temporal variability and non-parametric analysis. The results highlighted two contrasting accumulation regimes. The essential macroelements revealed a dynamic uptake pattern driven by the physiological demand, soil availability, and fertilization. K exhibited the highest transfer capacity, while Ca had a restricted translocation to the fruits, due to the intrinsic transport limitations. On the other hand, Li and Sr revealed a constrained accumulation, characterized by low concentrations, weak responsiveness to fertilization, and a strong dependence on the soil geochemical background and interannual dilution processes. The spatial variability between the cultivation sites and year-to-year changes in the dilution intensity was evidenced as the dominant driver of the transfer efficiency, while the varietal differences had a secondary but detectable role, mainly for the Ca–Sr discrimination. Overall, the results evidenced that the multielement accumulation in the raspberries was governed by the interplay between the soil geochemistry, physiological transport constraints, and environmental variability. Furthermore, the research provided a field-based, multiyear evidence supporting improved soil management, cultivar selection, as well as the strategies that may increase the fruit nutritional quality while minimizing the trace element risks.

## 1. Introduction

In recent decades, the areas intensively cultivated with raspberries have expanded rapidly in the world, mainly due to the growing demand for fruits with attractive sensory properties and recognised nutritional value. Raspberry (*Rubus idaeus* L.), often referred to as the “red jewel of the forest,” is the fruit of a perennial shrub belonging to the Rosaceae family [1], making it a botanical cousin of blackberries, strawberries, and roses. With its vibrant red hue, sweet-tart flavour, and intense aroma, raspberry has captivated human taste for millennia. While red-fruited types dominate global markets, cultivars with yellow, black, and purple fruits also exist, reflecting a broad genetic diversity within the species. Beyond culinary value, raspberry contain a wide range of bioactive constituents, particularly phenolic compounds such as ellagic acid, anthocyanins, and quercetin and polyphenols including flavonoids, tannins, and resveratrol. Other phytochemical classes, including carotenoids (e.g., lutein/zeaxanthin and β-carotene), are also present, typically at lower levels than polyphenols [2]. These phytochemicals have been extensively studied for their antioxidant, anti-inflammatory, and anticancer activities, as well as their potential roles in supporting cardiovascular, metabolic, and cognitive health.

Alongside these secondary metabolites, raspberries provide essential macro- and micronutrients, including calcium, magnesium, sodium, and potassium, which are indispensable for achieving optimal dietary balance. Increasingly, attention has also turned to the role of trace elements, which, though present in small amounts, play a vital role in biological and environmental systems. Trace elements not only contribute to plant metabolism but also influence human health through dietary intake, thereby linking agricultural practices, environmental conditions, and nutritional quality into a unified framework.

Romania has experienced a notable revival in raspberry cultivation during the last decade. In 2023, the total cultivated area was estimated at approximately 190 hectares, with a production volume of around 430 tons [3]. Compared to 2022, when production reached only 170 tons on a similar surface, this represents a more than 150% increase in total production, demonstrating both the adaptability of the species and the growing interest among farmers in expanding berry production.

The cultivation of raspberries is concentrated in areas with favourable pedoclimatic conditions, particularly in Transylvania, Moldova, and parts of northern Romania. These regions are characterized by moderate summers, sufficient rainfall, and fertile soils, which together provide optimal growth conditions for raspberries. Both conventional and organic systems are present, with organic raspberry production gaining popularity due to higher market value and consumer demand for pesticide-free fruits. However, comparative studies indicate that differences between organic and conventional practices in phenolic composition and antioxidant metrics are variable and frequently cultivar- and season-dependent, rather than consistently higher under organic management [4].

Future opportunities for raspberry production involve improved cultivation technologies, innovative processing, and market diversification. Moreover, advancing research on mineral nutrition and trace element dynamics (Li, Sr, Ca, Mg, Na, K), together with targeted biofortification strategies, may offer potential to simultaneously improve yield and fruit nutritional quality, thereby linking agricultural productivity with public health benefits. Because Li and Sr are not classical essential nutrients, such approaches must be framed cautiously, emphasising controlled management and evidence-based evaluation under agronomically realistic. In addition, risk-focused frameworks are well established for other trace metals in foods (e.g., cadmium), underscoring the importance of monitoring element occurrence in edible crops [5].

The inclusion of both essential macroelements (Ca, Mg, Na, K) and non-essential trace elements (Li and Sr) within a unified analytical framework may provide a comparative perspective on the plant uptake mechanisms. While macroelements are actively regulated and reflect physiological demand, trace elements such as Li and Sr are mainly governed by passive uptake processes or soil geochemical background. The integrated evaluation of these elements may allow a more comprehensive understanding of the balance between the nutritional accumulation and the environmental exposure mechanisms in the raspberry production systems.

Romania’s agricultural landscapes are characterized by various soil types, ranging from fertile chernozems to more acidic podzols, each influencing mineral bioavailability differently. In this context, trace elements such as lithium (Li) and strontium (Sr) can accumulate in edible crops not only through natural geochemical processes but also because of anthropogenic activities, including fertilizer application, industrial emissions, and waste amendments. Monitoring these elements in fruit crops like raspberry is crucial, since they serve as a direct dietary exposure pathway for consumers.

In Romanian food-chain assessments, Li has been quantified in foods from the national market, supporting the relevance of dietary intake considerations [6]. At the plant–soil interface, Li mobility and uptake are strongly modulated by soil characteristics (including pH, texture, mineralogy, and competing ions) and may vary among plant species and growing conditions. Soil mineralogy, mainly the presence of Li-rich clays and mica, denotes an important natural reservoir contributing to the plant uptake. Comparative assessments of the local cultivars indicate that two of them, Opal and Delniwa, differ in their Li uptake efficiency, with Opal exhibiting a higher capacity for root-to-fruit translocation [7]. These observations emphasize the role of the genotype-dependent mechanisms in regulating the Li dynamics and underline the importance of considering the cultivar selection in the trace element monitoring and management strategies [8].

On the other hand, Sr may behave as a chemical analogue of calcium (Ca) and can be absorbed through similar transport routes. In the Romanian soils, its availability is mainly related with the carbonate-rich substrates and, to a lesser extent, with the fertilizer-derived inputs. The longitudinal monitoring over two growing seasons showed that Sr may have a low phloem mobility, conducting to a preferential accumulation in roots and vegetative tissues, only small amounts being translocated to the fruit pulp [9]. This restricted redistribution within the plant can explain its general low contribution to the fruit mineral composition [10].

Therefore, the uptake, translocation, and accumulation of Li and Sr are processes of both agronomic and nutritional relevance. A controlled management of these elements may support biofortification protocols by enriching foods with potential beneficial trace elements. In contrast, an uncontrolled accumulation may rise risks to the food safety, mainly through the interference with Ca homeostasis or chronic Li exposure in humans [11,12,13].

Despite the recognized nutritional importance of the raspberries [3,14,15], multiyear, field-based research integrating both macroelements and non-essential trace elements such as Li and Sr remains scarce. This gap restricts the understanding of the soil–plant transfer dynamics under real agronomic conditions and limits the development of some evidence-based protocols for the nutrient management and food safety assessment. Despite the increasing importance of raspberries as nutritionally valuable fruit crops, comprehensive knowledge of the soil–plant transfer dynamics of both essential and non-essential elements remains limited, mainly under real field conditions and across multiple growing seasons [16]. Most previous studies have focused either on individual nutrients or on short-term experimental settings, without integrating macroelements and trace elements within a unified framework. The present study addressed this gap by providing a multiyear, multielement assessment of soil–fruit transfer processes in raspberry (*Rubus idaeus* L.), simultaneously evaluating essential macroelements (Ca, Mg, Na, and K) and trace elements (Li and Sr) across contrasting agroecosystems and cultivation practices. By combining transfer factor analysis, spatial–temporal variability survey, and statistical integration of soil buffering and dilution effects, this work provided new insights into the mechanisms governing element uptake, translocation, and accumulation in berry crops. The findings can contribute to a deeper understanding of how the soil geochemistry, environmental variability, as well as the cultivar-specific selectivity interact to shape fruit mineral composition, thereby supporting the development of sustainable fertilization protocols, improved cultivar selection, and evidence-based procedures to food safety monitoring and biofortification.

## 2. Results and Discussion

The mineral analysis of raspberry fruits revealed significant differences between the two studied cultivars, Opal and Delniwa, both in terms of essential macronutrients, calcium (Ca), magnesium (Mg), sodium (Na), and potassium (K), and in non-essential trace elements, namely lithium (Li) and strontium (Sr). The results, expressed in mg/kg dry weight, represent mean values calculated over a two-year monitoring period and provide insight into the cultivar-dependent mineral uptake, translocation, and accumulation patterns under natural and fertilized Romanian soil conditions. Given the multifactorial design of the study (variety × region × fertilization × year), the complete numerical datasets are further presented in Table 1, Table 2 and Table 3 to allow direct comparison across all experimental conditions. The following sections focus on the most relevant trends and comparative patterns, rather than on individual numerical values.

### 2.1. Essential Elements in Soil and Raspberry Fruits

The soils from the two investigated regions exhibited marked differences in both total trace element concentrations and exchangeable macronutrient pools (Table 1 and N–Soil-metal concentration).

Total lithium (Li) and strontium (Sr) concentrations were consistently higher in the Bucharest soils compared to those from Vâlcea, mainly for 2024, indicating a distinct geochemical background between the sites. When averaged across the two sampling years, Bucharest soils contained approximately 1.8-fold higher Li concentrations than the soils from Vâlcea, whereas Sr levels were of comparable magnitude, with slightly higher values in VâlceaVâlcea in 2025. Contrary, pronounced regional differences were observed for exchangeable macronutrients. Exchangeable calcium (Ca) concentrations were more than twofold higher in Bucharest soils than in Vâlcea, reflecting an increased base saturation and carbonate influence within the Bucharest area. Exchangeable magnesium (Mg) presented an opposite trend, with higher concentrations in Vâlcea soils compared to Bucharest, suggesting variations in parent material or long-term fertilization history. Exchangeable sodium (Na) and potassium (K) also varied considerably between regions and years. Bucharest soils exhibited moderate exchangeable Na contents, with a strong interannual decrease from 2024 to 2025, while Vâlcea soils maintained consistently higher Na levels. Exchangeable K concentrations were higher in Vâlcea, exceeding those measured in Bucharest by approximately 70%, indicating a more K-rich exchange complex in the Vâlcea soils.

The elemental composition of raspberry fruits varied markedly with variety, region, and fertilization regime (Table 2 and Supplementary Material S2–Raspberry-metal concentration).

Across all treatments, potassium (K) was the dominant macronutrient in the fruits, with mean concentrations ranging from approximately 1022 to 2970 mg/kg, confirming its central role in raspberries physiology. Magnesium (Mg) and calcium (Ca) followed in abundance, whereas sodium (Na), strontium (Sr), and lithium (Li) were present at substantially lower concentrations.

Clear regional differences were observed between Bucharest and Vâlcea. For both OPAL and DELNIWA varieties, fruits harvested from Vâlcea generally had higher Mg concentrations, mainly under fertilized conditions, while Bucharest fruits frequently displayed higher Na levels, particularly in the unfertilized plots. This pattern corresponded to the observed contrasts in the soil exchangeable nutrient availability between regions and indicated a strong soil influence on fruit mineral composition.

Fertilization effects were element-specific and variety-dependent. In DELNIWA, fertilization was associated with increased Ca and Mg concentrations in fruits from Vâlcea, whereas in Bucharest the same treatment tended to reduce Na and K concentrations, suggesting a dilution effect or enhanced ionic regulation under improved nutrient supply. In the OPAL variety, the fertilization consistently increased Mg concentrations in both regions, while Ca and K responses were more variable, reflecting cultivar-specific uptake and translocation capacities. Among trace elements, Sr concentrations exhibited moderate variability, with higher mean values observed in Vâlcea, whereas Li concentrations remained very low across all treatments and varieties. The limited accumulation of Li, coupled with its relatively high variability, indicated non-selective and weakly regulated uptake processes in raspberry fruits.

### 2.2. Transfer Factors

The soil–fruit transfer factors (TFs) for Li, Sr, Ca, Mg, Na, and K across varieties, regions, and fertilization regimes are summarized in Table 3.

Across all sites, varieties, and fertilization regimes, a consistent hierarchy of soil–fruit transfer factors was observed, with K having the highest values, followed by Mg, while Na and Sr revealed intermediate behaviour. Ca had a limited transfer, and Li consistently showed the lowest values.

Increased TF_Ca_ values were observed in the soils with lower exchangeable Ca content, suggesting a concentration-driven uptake response. Nevertheless, fertilization had a minimum effect on the Ca accumulation in fruits, reinforcing the hypothesis that the Ca transport is mainly governed by the physiological limitations rather than the soil availability. Consequently, the raspberry fruits acted as weak Ca sinks, in agreement with previous reports for berry crops [17,18,19].

Mg exhibited moderate to high TF values (0.26–0.93), with higher values frequently observed under fertilized conditions (e.g., up to 0.93 in OPAL, Bucharest). As a mobile element and a key component of chlorophyll, Mg was efficiently absorbed and translocated within the plant [20,21]. TF values approaching unity indicated a tendency toward an enhanced uptake under favourable conditions, evidencing the importance of Mg availability for improving fruit mineral quality.

Na exhibited moderate TF values (0.07–0.37), with sporadic higher values observed in soils with low exchangeable Na concentrations. Although Na is not an essential nutrient for raspberries, its uptake occurs via non-selective cation transport mechanism [22]. The lack of a consistent fertilization effect on TF_Na_ suggested an efficient physiological regulation, preventing excessive Na accumulation in the fruits [22]. Overall, raspberries exhibited a partial exclusion strategy toward Na.

K displayed by far the highest TF values (2.85–12.27), mainly in Bucharest for the 2024 year. As the predominant macronutrient in the raspberry fruits, it plays an essential role in the osmotic regulation, enzyme activation, as well as sugar transport [23]. Its high TF values reflected a strong physiological demand, efficient root uptake, and active translocation to fruits [24]. Although fertilization generally enhanced K uptake, the increased TFs were also observed under unfertilized conditions, indicating that K accumulation was intensively driven by intrinsic plant requirements rather than the soil abundance alone. These results confirmed the raspberries are strong K accumulators [23,24].

Li concentrations in raspberries were consistently low, resulting in TF values having a generally trend below 0.1 value. Sporadically higher TF (up to 0.20), observed mainly in 2025 within Bucharest samples, was associated with low soil Li concentration, evidencing a dilution effect rather than active accumulation [25]. Li has no known biological function in higher plants, and its accumulation is generally dominated by non-active uptake processes, highlighting limited physiological control [25,26]. Therefore, soil availability and ionic competition were considered as factors that mainly governed the Li accumulation in raspberries. No consistent varietal differences were observed, suggesting a limited role of genotype in Li uptake [26]. Overall, raspberries may be considered as effective Li excluders [25].

Sr presented moderate TF values, typically ranging from 0.21 to 0.58, with slightly higher values in Bucharest soils compared to Vâlcea. Given the chemical similarity between Sr and Ca, Sr uptake is closely associated with the Ca transport mechanism rather than the selective accumulation mechanisms [25]. The TF values indicated a moderate mobility of Sr within the soil–plant system, mainly in the soils characterized by a lower Ca/Sr ratio. No evidence of excessive Sr accumulation in fruits was detected, confirming that the Sr uptake in raspberries remained physiologically constrained [27].

### 2.3. Influence of Site, Variety, and Fertilization

Site-specific effects: Raspberries grown in Bucharest soils generally exhibited higher TFs for Mg, Na, and K compared to those cultivated in Vâlcea. This pattern was attributed to lower exchangeable nutrient pools and increased relative bioavailability in Bucharest soils, whereas Vâlcea soils displayed more buffered chemical characteristics and more stable TF values.

Varietal effects: Differences between the OPAL and DELNIWA cultivars were relatively minor. DELNIWA occasionally exhibited slightly higher TFs for Mg and K, suggesting marginally enhanced nutrient translocation to fruits; nevertheless, these differences were not systematic across sites or years.

Fertilization effects: Fertilization had the strongest impact on Mg and K uptake, leading to increased fruit concentrations and higher TF values. Contrary, Ca transfer remained generally unaffected by fertilization, evidencing intrinsic transport limitations. Uptake of the trace elements (Li and Sr) showed no consistent response to fertilization.

It was generally observed that, across all sites, varieties, and treatments, transfer factors exhibited a consistent hierarchical pattern, with K showing by far the highest values, followed by Mg, while Na and Sr displayed similar intermediate transfer. Ca exhibited lower transfer efficiency, and Li consistently presented the lowest one. Overall, the general sequence can be expressed as: K ≫ Mg > Na ≈ Sr > Ca ≫ Li. This hierarchy reflected both the physiological demand of raspberries and the chemical behaviour of the elements in soil–plant systems.

These results demonstrated that the mineral composition of raspberries was primarily driven by element mobility and physiological demand rather than the total soil concentrations. High TFs for K and Mg reflected active accumulation processes, whereas low TFs for Ca, Li, and Sr indicated restricted transport or passive uptake pathway. From both agronomic and food safety perspectives, no evidence of excessive trace element accumulation was observed. In this respect, fertilization strategies should prioritize balanced K and Mg supply, while recognizing the inherent limitations associated with Ca enrichment in raspberry fruits.

The observed patterns were consistent with the findings reported for other berry crops, such as strawberries and blueberries, where the element accumulation was similarly governed by the physiological demand and by the element mobility [17,18,19,23,24]. In these cases, K was typically the dominant macronutrient, with a high accumulation and efficient translocation to the fruits, while Ca exhibited restricted mobility and limited transfer due to its low phloem transport capacity [17,18,19]. Mg generally had an intermediate behaviour, evidencing its role in the chlorophyll structure and metabolic activity [20,21].

Regarding the trace elements, previous research on berry crops has also reported low and variable accumulation, mainly governed by the soil geochemical background rather than by an active physiological regulation [25,26,27]. These crops tended to have limited translocation of the elements such as Sr, while Li accumulation remained low and strongly dependent on the soil availability [25,26]. Overall, these cross-species similarities sustained the observation that the element-specific mobility and plant physiological constraints represented the main drivers of the mineral composition in the berry fruits.

### 2.4. Statistical Analysis

**Figure 1:**
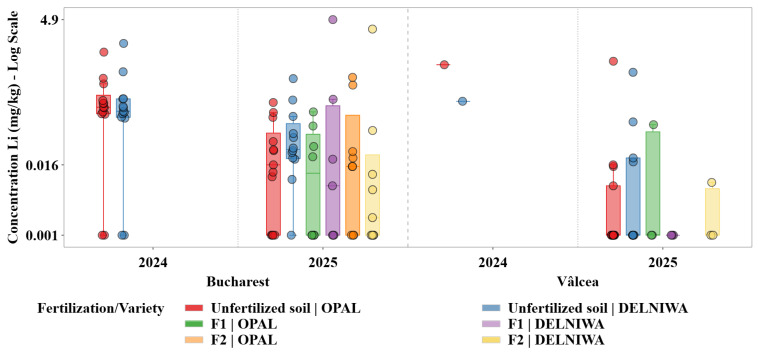
Li concentration—Bucharest vs. Vâlcea (Year × Fertilization × Variety).

**Figure 2:**
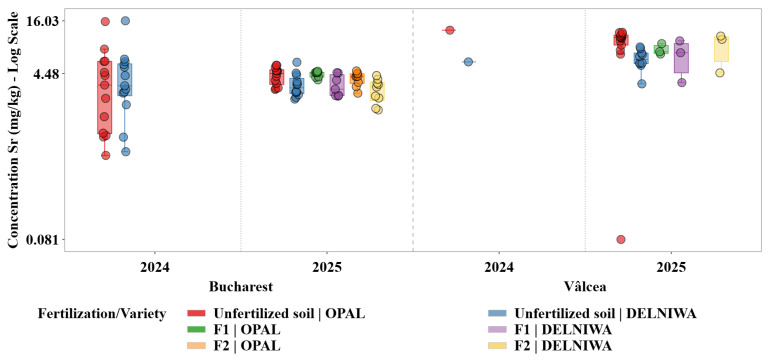
Sr concentration—Bucharest vs. Vâlcea (Year × Fertilization × Variety).

**Figure 3:**
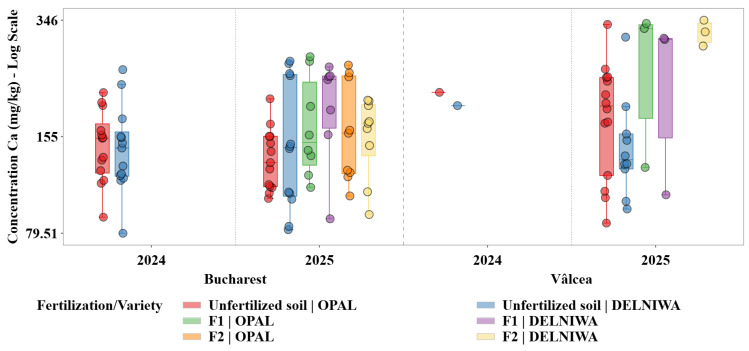
Ca concentration—Bucharest vs. Vâlcea (Year × Fertilization × Variety).

**Figure 4:**
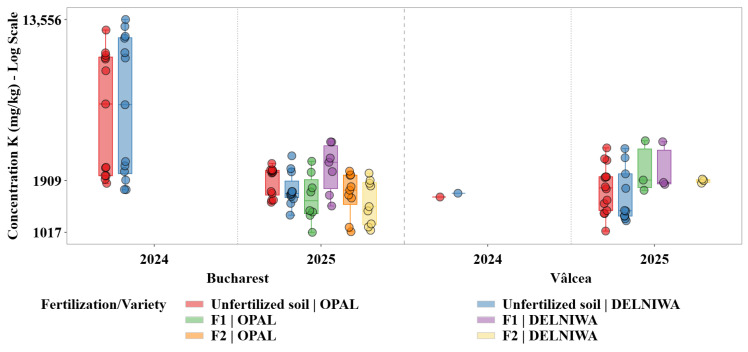
K concentration—Bucharest vs. Vâlcea (Year × Fertilization × Variety).

**Figure 5:**
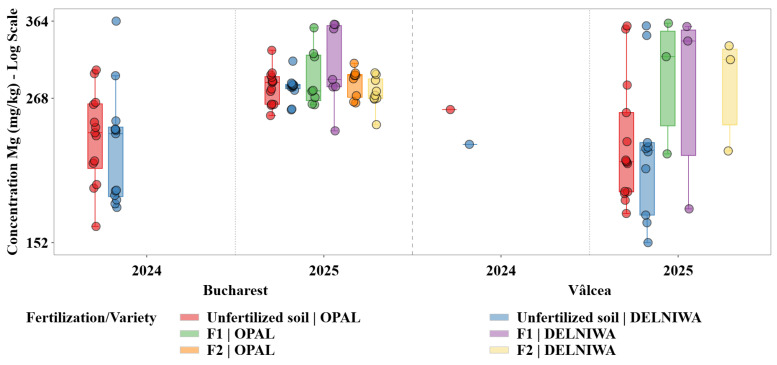
Mg concentration—Bucharest vs. Vâlcea (Year × Fertilization × Variety).

**Figure 6:**
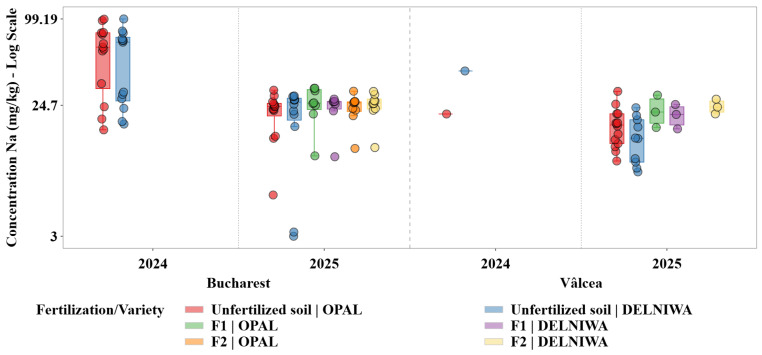
Na concentration—Bucharest vs. Vâlcea (Year × Fertilization × Variety).

Descriptive statistical analysis was used as the primary exploratory tool to characterize the distributions of metal concentrations across combinations of cultivation site, sampling year, fertilization regime, and raspberry variety. For each metal, grouped statistics were computed at the level of location × year × fertilization × variety, including sample size (N), minimum and maximum values, arithmetic mean, median, first and third quartiles (Q1 and Q3), interquartile range (IQR), and standard deviation. The median and IQR were evidenced as robust measures of central tendency and dispersion, given the pronounced skewness and heteroscedasticity observed in several metal distributions (Figure 1, Figure 2, Figure 3, Figure 4, Figure 5 and Figure 6). Graphical representations were designed as grouped boxplots overlaid with individual data points, allowing simultaneous visualization of distributional structure and sample-level variability. The *x*-axis was hierarchically structured to reflect the combined effects of locality and sampling year, while fertilization–variety combinations were encoded through colour mapping. To accommodate the wide dynamic range of concentrations, mainly for Li and alkali metals, all plots were displayed on a logarithmic *y*-axis. Vertical dashed separators were introduced to visually delineate transitions between years and locations, thereby facilitating direct intra- and inter-site comparisons.

This combined statistical–visual framework provided a transparent and reproducible basis for identifying systematic patterns, temporal shifts and treatment-specific effects in raspberries metal composition.

Figure 1 illustrates the distribution of lithium concentrations in raspberries across sites, years, fertilization regimes, and varieties.

Li concentrations were consistently low and strongly right-skewed at both sites, with higher median values and higher dispersion in Bucharest compared to Vâlcea, indicating a dominant control of background soil geochemistry rather than agronomic management. Consistent with this assumption, median lithium concentrations generally remained well below 0.16 mg/kg across both sites and years, with strongly right-skewed distributions and occasional high-end outliers. The pronounced reduction in median values observed in 2025 relative to 2024, particularly in Bucharest, indicates that interannual dilution-related processes exert a dominant control over effective lithium transfer to fruit tissues.

Sr was included in the analysis as a geochemically conservative trace element, expected to exhibit limited physiological regulation and to reflect site-specific soil background conditions rather than agronomic management (Figure 2).

Sr concentrations displayed relatively narrow interquartile ranges, with median values typically clustering around 3.0–4.6 mg/kg across both years. In 2024, under unfertilized conditions, median values were about 3.33 mg/kg for DELNIWA and 3.39 mg/kg for OPAL. Systematic differences between Bucharest and Vâlcea were observed, while interannual shifts remained modest compared to Li, supporting the idea that Sr uptake was a predominantly passive process constrained by site-specific soil background.

As shown in Figure 3, Ca concentrations in raspberries reflected an actively regulated macroelement involved in structural and signalling functions, with accumulation patterns clearly distinct from those of trace elements.

Consistent with this role, median Ca concentrations in raspberry fruits were one to two orders of magnitude higher than those of Li and Sr. In Bucharest, median Ca concentrations generally ranged between 129 and 170 mg/kg, depending on the year and the fertilization treatment. For Vâlcea, Ca concentrations were higher and more variable. In 2025, median values reached about 303 mg/kg for DELNIWA under F1 fertilization and about 327 mg/kg for OPAL, under the same treatment. Between 2024 and 2025, both median values and distribution width showed measurable shifts, mainly for the Bucharest site, where changes in Q1–Q3 spread indicated enhanced interannual variability and a dynamic accumulation regime modulated by environmental conditions rather than by static soil background alone.

Figure 4 illustrates that K concentrations in raspberries occurred at substantially higher levels than other macroelements, reflecting high physiological demand combined with tight homeostatic regulation.

K concentrations displayed high absolute levels, with median values typically in the range of 900–1500 mg/kg and interquartile intervals commonly spanning 700–1800 mg/kg, depending on site and year. Despite this wide concentration range, interannual shifts between 2024 and 2025 are relatively modest compared to Ca and Mg, indicating a strongly regulated accumulation regime in which dispersion reflects site-specific availability rather than pronounced year-to-year variability.

Figure 5 highlights Mg as an essential macroelement exhibiting active accumulation, with concentration patterns that are broadly comparable to Ca but displaying higher sensitivity to interannual changes in soil availability and environmental conditions.

Mg concentrations reflected an actively regulated macroelement, with distribution patterns comparable in magnitude to Ca. For Bucharest, the median Mg concentrations generally ranged between 233 and 293 mg/kg, depending on the fertilization treatment. At the Vâlcea site, Mg exhibited a higher variability, with median values reaching approximately 316–336 mg/kg, under fertilized treatments for the 2025 year.

The distribution presented in Figure 6 indicates that Na accumulation in raspberry fruits followed a pattern distinct from both K and Mg, consistent with a weakly regulated macroelement mainly influenced by site-specific soil conditions.

Na concentrations exhibited moderate absolute levels. In Bucharest during 2024, without fertilization, the median values reached approximately 63–68 mg/kg, whereas in 2025 the median values considerably decreased, ranging between 24 and 26 mg/kg. Compared to K and Mg, Na showed increased dispersion and more pronounced site-dependent variability, mainly for the Bucharest site, indicating limited homeostatic control and enhanced sensitivity to heterogeneous soil environments.

The hierarchical structuring of the boxplot representations allowed across all metals a clear discrimination between the effects of sampling year, cultivation site, fertilization regime, and variety, while the logarithmic scale facilitated comparison across elements spanning several orders of magnitude in concentration.

Ca, K, Mg, and Na exhibited distinct yet partially overlapping accumulation patterns, which can be attributed to their essential physiological roles and to the existence of active uptake and translocation mechanisms regulating their transport from soil to fruit tissues. Therefore, variations in their concentrations reflected a dynamic interplay between soil availability, fertilization regime, and plant nutritional demand. In contrast, Li and Sr, classified as trace elements in the present study, were detected at substantially lower concentrations and showed markedly different accumulation behaviour. Their distributions were characterized by limited variability and pronounced right skewness, indicating predominantly passive uptake processes controlled by background soil geochemistry rather than by plant physiological regulation. Li displayed median concentrations consistently located near the lower bound of the logarithmic scale, supporting the hypothesis that its accumulation in raspberries was strongly constrained by soil background levels and exhibits minimal responsiveness to agronomic inputs [13].

Important temporal variability was observed across all investigated macroelements, with several elements exhibiting increased dispersion and, in some cases, higher median concentrations in 2025 relative to 2024. This pattern was evident for Mg and Ca, indicating an enhanced sensitivity of these elements to interannual fluctuations in soil nutrient availability and prevailing climatic conditions. Such variability suggested that changes in soil moisture regime, temperature, and nutrient mobility between growing seasons may have exerted a significant influence on their uptake dynamics [28]. Contrary, K and Na displayed comparatively stable median concentrations across the two sampling years, although noticeable variations in distribution width point to year-dependent heterogeneity in their bioavailability rather than to systematic shifts in accumulation levels.

Temporal trends for trace elements followed a distinct trajectory. Li, and to a similar extent Sr, exhibited systematically higher concentrations in 2024 compared to 2025, particularly for the Bucharest site. The downward shift observed in 2025 was accompanied by a marked reduction in dispersion, indicating a more uniform and constrained uptake regime during the later sampling year. This behaviour supported the hypothesis that the accumulation of these trace elements was strongly governed by interannual variations in soil geochemical background rather than by physiological demand or management practices [29].

Clear spatial differentiation between the two cultivation sites emerged as a dominant factor influencing metal accumulation patterns. For macroelements, Bucharest generally exhibited higher median concentrations and broader interquartile ranges for K and Na, reflecting a more heterogeneous soil environment and higher variability in nutrient availability. Vâlcea, in contrast, was characterized by narrower distributions for several macroelements, suggesting a more homogeneous soil background and more stable uptake conditions.

The spatial contrasts were even more pronounced for the trace elements. Li and Sr concentrations were consistently higher in Bucharest than in Vâlcea across both sampling years, while Vâlcea samples frequently clustered near the lower end of the logarithmic scale. The narrow dispersion observed in Vâlcea indicated a geochemically constrained system in which soil background levels imposed a strong upper limit on plant uptake, effectively overriding potential influences of fertilization or varietal differences [11].

Fertilization effects differed substantially between macroelements and trace elements. For Ca and Mg, fertilized treatments (F2 and F1) were associated with higher median concentrations and, in some cases, expanded interquartile ranges compared to unfertilized controls. This response reflected the essential role of these elements in plant metabolism and the presence of active uptake and translocation mechanisms that allowed plants to capitalize on increased soil availability.

Contrary, K and Na showed weaker and less consistent responses to fertilization, suggesting that their accumulation is more tightly regulated by site-specific soil properties and intrinsic ionic balance than by short-term nutrient inputs [30].

Trace elements displayed minimal and inconsistent responses to fertilization. Li and Sr concentrations frequently overlapped with those observed under unfertilized conditions, indicating that fertilization did not substantially alter their uptake. This finding reinforced the fact that these elements were mainly absorbed through passive processes and that their accumulation was largely decoupled from agronomic nutrient management [31].

Varietal differences in metal accumulation were generally secondary to temporal and spatial effects but remained detectable for certain elements. Among the macroelements, OPAL and DELNIWA typically exhibited comparable median concentrations. These patterns suggested varietal modulation of uptake efficiency rather than fundamentally different accumulation strategies.

For the trace elements, varietal effects were weak and non-systematic. Differences between varieties were often within the interquartile range of the distributions and did not consistently persist across years or sites. This limited varietal differentiation further indicated that the genetic control over Li and Sr uptake was minor compared to the constraints imposed by soil geochemical background [11].

Taken together, these results highlighted the existence of two contrasting accumulation regimes in raspberry fruits. Macroelements exhibited dynamic and responsive behaviour, driven by a combination of soil availability, fertilization, and physiological demand, resulting in pronounced temporal and spatial variability. Contrary, trace elements such as Li and Sr revealed a constrained transfer pattern that appeared to be strongly influenced by the soil background composition, with a limited observable response to fertilization under the present conditions. Nevertheless, this may also reflect the absence or low levels of these elements in the applied fertilizers.

**Figure 7:**
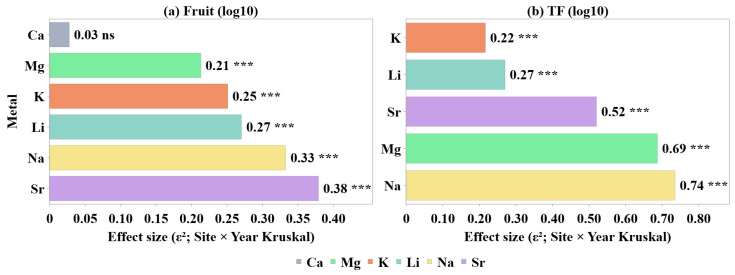
Site × Year effect sizes (Kruskal ε^2^)—(**a**) Fruit Concentration (log10), (**b**) TF (log10). *** *p* < 0.001. ns = not significant effect.

To quantify the combined spatial and interannual variability observed within Figure 1, Figure 2, Figure 3, Figure 4, Figure 5 and Figure 6, non-parametric Kruskal–Wallis tests were applied. Figure 7 summarizes the magnitude of the Site × Year interaction effects, quantified using the Kruskal–Wallis test and expressed as epsilon squared (ε^2^), for both fruit metal concentrations (log10) and transfer factors (TF, log10).

Figure 7a summarizes the magnitude of Site × Year interaction effects on raspberry metal concentrations expressed on a logarithmic scale. Effect sizes varied among elements, with Sr, Na and K showing the strongest sensitivity to combined spatial and interannual variability, whereas Ca exhibits only weak Site × Year dependence, consistent with its more stable accumulation behaviour. For the fruit concentrations, the strength of the Site × Year interaction substantially varied among metals. The largest effect sizes were observed for Sr (ε^2^ ≈ 0.38) and Na (ε^2^ ≈ 0.33), followed by K (ε^2^ ≈ 0.25), Li (ε^2^ ≈ 0.27) and Mg (ε^2^ ≈ 0.21). In contrast, Ca exhibited a very small effect size (ε^2^ ≈ 0.03), indicating that its fruit concentration was only weakly influenced by the combined effect of site and year.

Figure 7b shows the corresponding Site × Year effect sizes for the transfer factors (TF). Across all elements, ε^2^ values were systematically higher than those observed for the fruit concentrations, indicating that soil-to-fruit transfer efficiency integrated spatial and interannual variability more strongly than the absolute tissue concentrations, thereby amplifying site- and year-dependent differences. The strongest Site × Year effects were observed for Na (ε^2^ ≈ 0.74) and Mg (ε^2^ ≈ 0.69), followed by Sr (ε^2^ ≈ 0.52). Li (ε^2^ ≈ 0.27) and K (ε^2^ ≈ 0.22) exhibited comparatively lower, yet still substantial, interaction effects, indicating that their soil-to-fruit transfer was less influenced by the combined spatial and temporal variability. The amplification of Site × Year effects at the TF level indicated that interannual changes in soil conditions and site-specific characteristics more strongly propagated through transfer processes than through the fruit concentrations alone. This highlighted TF as a sensitive indicator of the environmental forcing acting on metal uptake dynamics. This contrast indicated that the transfer processes integrated spatial and temporal variability more strongly than the absolute fruit concentrations, acting as a sensitive indicator of site-specific and interannual changes in soil–plant transfer dynamics.

**Figure 8:**
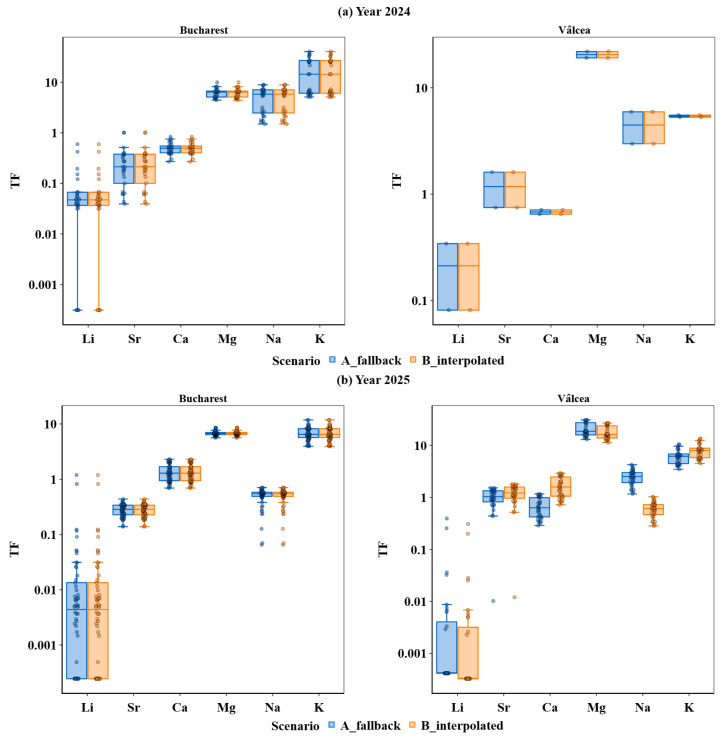
Comparison of transfer factor (TF) distributions obtained using two alternative soil reference scenarios: Scenario A (measured soil background) and Scenario B (trend-based interpolated soil concentrations)—(**a**) Year 2024 and (**b**) Year 2025.

Given the pronounced Site × Year interaction effects identified across several metals, it was necessary to verify that these patterns were not artefacts arising from soil reference selection. To this end, two alternative soil background scenarios were evaluated to assess the robustness of transfer factor (TF) estimates (Figure 8).

To evaluate the sensitivity of transfer factor (TF) estimated for the selected soil reference, the TF distributions derived from measured soil background concentrations (Scenario A) were compared with those based on trend-interpolated soil concentrations (Scenario B) for the 2024 and 2025 harvests (Figure 8a,b). Across both years, TF distributions derived under the two soil reference scenarios showed a high degree of overlap, with median values differing only marginally and remaining well within the corresponding interquartile ranges. Scenario-dependent differences were consistently smaller than the intrinsic within-group variability associated with the site, year, and fertilization regime, indicating that the absence of direct soil measurements for Vâlcea in 2024 did not introduce a material bias in transfer estimates.

For the Vâlcea site, Scenario A was based on measured soil concentrations from 2023, whereas Scenario B relied on a trend-based interpolation of soil values for 2024. Across all investigated metals, TF distributions obtained under the two scenarios displayed a high degree of overlap and comparable central tendencies, with no systematic shifts in median values or dispersion.

Trace elements (Li and Sr) exhibited tight clustering under both scenarios, indicating that their transfer was weakly sensitive to the assumed soil reference and was primarily governed by background soil geochemistry. For the macroelements (Ca, Mg, K and Na), slightly increased dispersion was observed, consistent with their higher mobility and broader concentration ranges; nevertheless, the relative ordering of metals and the magnitude of TF values remained unchanged.

Differences between Scenario A and Scenario B were consistently smaller than the within-group variability associated with site, year, and fertilization regime. This confirmed that the absence of the direct soil measurements for Vâlcea in 2024 did not introduce a significant bias into the estimation of transfer factors. Therefore, Scenario A was retained as the main reference framework for subsequent analysis, while Scenario B was used exclusively for sensitivity testing.

Given the strong concordance between the two soil reference scenarios, the observed transfer patterns were more plausibly attributed to inherent soil–plant transfer mechanisms rather than to methodological artefacts arising from the selection of soil background values.

**Figure 9:**
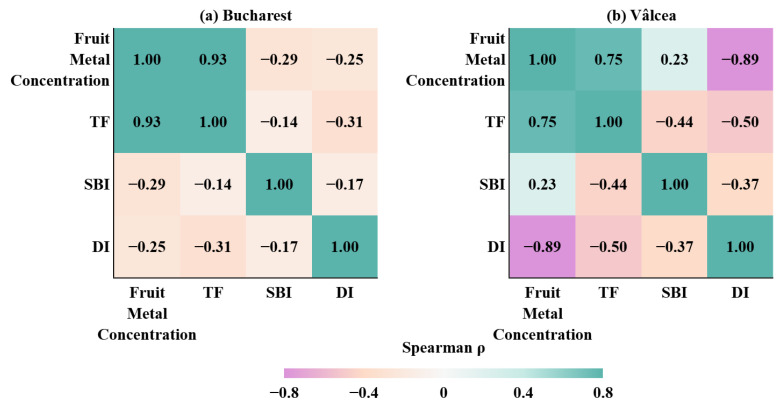
Spearman correlation matrix (TF–SBI–DI integration)—(**a**) Bucharest, (**b**) Vâlcea.

On this validated basis, correlation analysis was applied to integrate transfer (TF), soil background (SBI) and dilution intensity (DI) into a unified associative framework, allowing site-specific drivers of metal transfer variability to be explored (Figure 9).

The two correlation matrices revealed contrasting site-specific interaction structures: Bucharest (Figure 9a) exhibited a multi-driver regime characterized by weak to moderate associations among TF, SBI and DI, whereas Vâlcea (Figure 9b) displayed a more coherent, dilution-dominated structure marked by strong negative correlations between DI and both fruit concentrations and transfer factors. This contrast indicated that interannual variability in Bucharest arose from heterogeneous soil–plant interactions, while in Vâlcea it was primarily governed by dilution-related processes acting on a comparatively stable soil background.

At both sites, fruit metal concentrations exhibited a strong positive monotonic association with TF (ρ = 0.93 for Bucharest and ρ = 0.75 for Vâlcea), reflecting the intrinsic mathematical relationship between fruit concentrations and the transfer factor. This association was therefore considered structural and primarily descriptive, confirming internal consistency of the calculated indices rather than indicating a direct mechanistic control.

Beyond this expected relationship, clear site-specific differences emerge in the interaction between soil background and dilution effects. In Bucharest, SBI showed weak to moderate negative correlations with fruit concentrations (ρ = −0.29) and TF (ρ = −0.14), suggesting that higher baseline soil concentrations did not directly translate into increased accumulation in fruits. This pattern was consistent with the presence of uptake regulation mechanisms and heterogeneous soil–plant interactions in the Bucharest system. Dilution effects (DI) were only weakly negatively associated with fruit concentrations (ρ = −0.25) and TF (ρ = −0.31), indicating that interannual dilution contributed to variability, but did not dominate the accumulation regime.

Contrary, the Vâlcea site exhibited a different correlation structure. Fruit concentrations were strongly and negatively associated with DI (ρ = −0.89), while moderate negative correlations were also observed between TF and DI (ρ = −0.50) and between SBI and DI (ρ = −0.37). This pattern indicated that dilution dynamics exerted a substantially stronger control on interannual variability of fruit metal concentrations in Vâlcea. The more coherent correlation structure suggested a comparative stable soil background, where fluctuations in effective availability were primarily governed by dilution-related processes rather than by absolute soil metal content.

The results indicated that, while transfer patterns at both sites were internally consistent, the dominant drivers of variability differed. Bucharest was characterized by a more complex, multi-driver regime involving soil heterogeneity and variable uptake constraints, whereas Vâlcea displayed a more constrained system in which dilution effects played a central role. These findings supported the site-specific interpretation of transfer processes and justified the subsequent stratified analysis by site, year, and fertilization regime.

**Figure 10:**
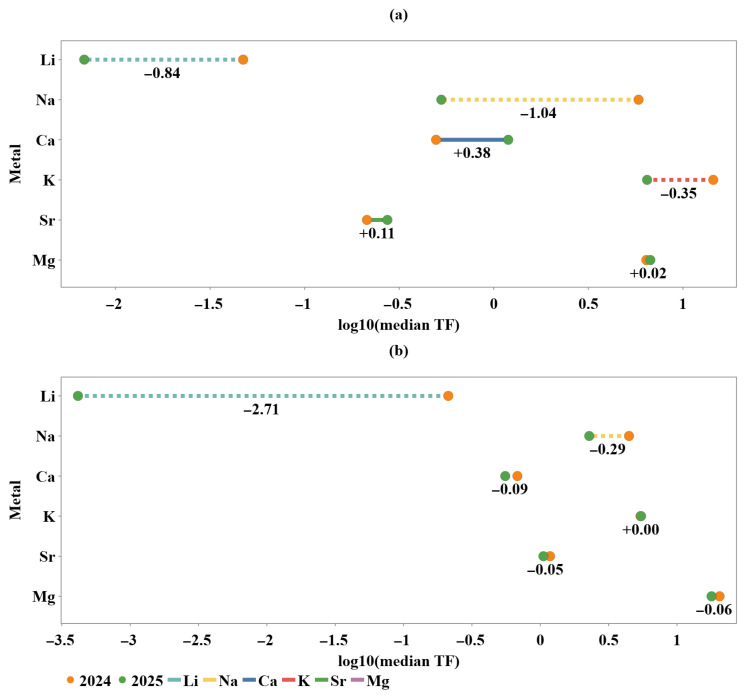
Interannual shift in transfer intensity (log10(median TF), 2024 → 2025): (**a**) Bucharest, (**b**) Vâlcea. Coloured line indicates metal; solid lines denote increases and dotted lines denote decreases.

While the Spearman correlation matrices integrated transfer (TF), soil buffering (SBI) and dilution intensity (DI) into a unified associative framework, they did not resolve how strongly these drivers translate into year-to-year changes in transfer efficiency. Interannual shifts in transfer intensity were therefore examined using metal-specific slopegraphs of log10(median TF), separately designed for Bucharest and Vâlcea (Figure 10).

The resulting patterns revealed site-dependent interannual transfer dynamics that were consistent with the dilution regimes inferred from DI. In Bucharest (Figure 10a), pronounced negative shifts were observed for Li and Na (Δlog10TF ≈ −0.84 and −1.04, respectively), indicating a strong reduction in effective soil-to-fruit transfer between 2024 and 2025. These metals are known to be highly responsive to changes in water availability and soil solution dilution, suggesting that increased dilution intensity in 2025 acted as a dominant limiting factor [32]. In contrast, Ca exhibited a clear increase in transfer intensity (Δlog10TF ≈ +0.38), while Sr and Mg remained near quasi-stationary conditions, suggesting partial decoupling from dilution-driven control and a stronger influence of soil buffering and physiological regulation. K showed an intermediate decline (Δlog10TF ≈ −0.35), reflecting mixed sensitivity to dilution and nutrient regulation processes.

In the Vâlcea region (Figure 10b), the interannual response was more coherent and dominated by a strong decrease in Li transfer (Δlog10TF ≈ −2.71), consistent with a system mainly governed by dilution intensity rather than heterogeneous uptake constraints. On the other hand, the remaining elements exhibited only minor interannual changes, with shifts close to zero for K, Ca, Sr, and Mg, and a modest decrease for Na (Δlog10TF ≈ −0.29). This supported the interpretation that Vâlcea as a dilution-controlled system with limited buffering-induced divergence. Taken together, these patterns confirmed that DI acted as a key modulator of interannual transfer variability, while its impact was filtered through site-specific buffering capacity and metal-dependent uptake behaviour.

Because fertilization regimes can modify soil buffering and nutrient availability [17] their potential role as modulators of interannual transfer variability was also examined. However, for F2 and F1 treatments, data were available for a single year only, precluding a quantitative assessment of interannual shifts under fertilized conditions. In both sites, fertilized treatments were characterized by reduced temporal variability relative to the Control, suggesting that fertilization may attenuate or mask interannual fluctuations in metal transfer through enhanced soil buffering capacity and stabilized nutrient availability. As a result, while dilution intensity emerged as a dominant driver of interannual transfer shifts under unfertilized conditions, its effect could not be quantitatively resolved under fertilized regimes, highlighting fertilization as a potential stabilizing modulator rather than a driver of variability.

Across all investigated elements, fruit concentrations displayed strong site- and year-dependent variability, with the macroelements (Ca, K, Mg, and Na) showing broader and more responsive distributions, consistent with active uptake and physiological regulation, whereas the trace elements (Li and Sr) exhibited constrained, right-skewed patterns indicative of passive uptake largely governed by background soil geochemistry.

Non-parametric effect-size analysis confirmed that Site × Year interaction effects were metal-dependent and substantially stronger for the transfer factors than for the fruit concentrations, indicating that the transfer efficiency integrated spatial and interannual variability more strongly than the absolute tissue concentrations. Na and Mg showed the highest Site × Year sensitivity at the TF level, while Ca remained comparatively stable at the fruit-concentration level.

A dedicated validation of soil reference scenarios demonstrated that the transfer estimation was robust to the choice of baseline soil assumption for Vâlcea, as scenario-dependent differences remained smaller than the intrinsic variability associated with site, year, and management. This supported the interpretation of the observed transfer patterns as reflecting intrinsic soil–plant transfer processes rather than methodological artefacts.

Correlation-based integration of TF, SBI, and DI revealed contrasting site-specific regimes: Bucharest exhibited a multi-driver structure consistent with heterogeneous soil–plant interactions and partial uptake regulation, whereas Vâlcea displayed a dilution-dominated structure, with DI acting as a key modulator of interannual variability. For this reason, slopegraph analysis of interannual shifts in the transfer intensity highlighted pronounced metal-specific decreases between 2024 and 2025, mainly for Li and Na, supporting a strong role of dilution-mediated forcing on transfer efficiency.

Finally, fertilization effects could not be evaluated in a fully interannual framework due to the single-year availability for the treatments. Nevertheless, reduced dispersion under fertilization suggested a stabilizing role through enhanced buffering rather than a primary driver of interannual change. Overall, the combined evidence indicated that transfer factors provided a sensitive lens for resolving site-specific and interannual controls on metal transfer in raspberry systems, with dilution intensity and soil background constraints jointly shaping metal-dependent accumulation regimes.

### 2.5. Overview on Li Uptake and Translocation

The detection of Li in raspberry fruits, even at trace concentrations, represents a noteworthy finding due to the element uncertain physiological role in plants and its emerging nutritional and toxicological relevance in humans. Although Li is not currently classified as an essential nutrient for higher plants, increasing evidence indicates that it can exert dual effects depending on concentration: at low doses, Li may function as a beneficial element by stimulating photosynthesis, stabilizing chlorophyll, and influencing auxin metabolism, while at elevated levels, it becomes detrimental, inducing oxidative stress, impairing enzymatic activity, and inhibiting plant growth.

The present study confirmed the presence of lithium in raspberry fruits grown under Romanian conditions, with measured concentrations ranging from 0.16 to 0.63 mg/kg dry weight. These values, though relatively low, are consistent with findings in other horticultural crops where Li has been shown to accumulate in edible tissues because of both natural soil content and anthropogenic influences [33]. The detected concentrations were low and consistent with the values reported for plant-derived foods [33]. Although no specific regulatory limits for Li in foods are currently established, the literature indicates that dietary Li intake typically occurs at trace levels and may contribute to the physiological effects at low doses [34]. In this context, the contribution from raspberry consumption under the present conditions can be considered negligible, and no immediate food safety concerns arise. Nevertheless, continued monitoring is required considering the variability of Li in the soils and its potential biological effects [11].

An interesting observation emerged when comparing cultivars, Delniwa consistently accumulating more Li in fruits compared to Opal. This suggested the existence of genotype-dependent mechanisms regulating root absorption and xylem–phloem transport of Li, potentially linked to differences in ion channel activity or root transporter selectivity [35]. Such variability highlighted the importance of considering genetic factors when evaluating the behaviour of non-essential elements in fruit crops, since cultivar choice can directly influence the extent of trace element accumulation in edible tissues [26].

From a nutritional perspective, the contribution of raspberries to dietary Li intake was modest, but not negligible. Recent studies have suggested that trace Li exposure, primarily through drinking water and plant-based foods, may exert neuroprotective and mood-stabilizing effects at low doses [36]. Thus, while the concentrations measured in this study did not pose a toxicological risk, they could still play a minor role in shaping functional food properties, mainly in the context of biofortification research aimed at enhancing the micronutrient profile of fruits.

Summarising, Li uptake and translocation in raspberries demonstrated both the plasticity of trace element behaviour in plants and the potential nutritional significance of trace concentrations. The observed cultivar differences further underscore the demand for integrated studies combining soil chemistry, plant physiology, and genetic factors to better understand how Li interacts with the mineral nutrition of fruit crops and how it may contribute to human health.

### 2.6. Sr Accumulation and Ca Interactions

The behaviour of Sr in plants is of particular interest due to its chemical analogy to Ca, sharing similar ionic radius and charge, which allows Sr^2+^ to enter plant cells via the same transport pathways as Ca^2+^ [37]. This structural similarity makes Sr a competitive substitute for Ca in both uptake and tissue distribution. Nevertheless, unlike Ca, Sr has low phloem mobility, which limits its redistribution to developing fruits and generally leads to preferential accumulation in roots and vegetative organs [38].

The soil analysis indicated relatively high concentrations of the exchangeable Sr, with values ranging between 7.25 and 22.95 mg/kg, mainly in the carbonate-rich soils typical for Romanian orchards. This background availability created the potential for Sr uptake into raspberry plants. Despite the favourable Ca/Sr ratios in soil (187.8–640.9), measurable amounts of Sr were detected in raspberries, demonstrating that the root uptake mechanisms do not completely discriminate between Ca and Sr [39].

In fruits, Sr concentrations were found in the range of 1.57–16.03 mg/kg, with Delniwa consistently exhibiting higher accumulation compared to Opal. This differential behaviour suggested genotypic variability in Ca transporters or exclusion mechanisms [40], with Opal displaying higher efficiency in favouring Ca over Sr during translocation.

The ratio of Ca to Sr provided valuable insight into the competitive dynamics of these ions. While soils maintained a strong Ca dominance (high Ca/Sr ratios), the values in fruits were significantly reduced, indicating partial co-transport of Sr into edible tissues [37]. Imore important, the Ca/Sr ratio was 106.6 in Opal and only 47.3 in Delniwa, highlighting that Opal maintained a stronger selective bias toward Ca accumulation, which directly supported fruit firmness and reduced Sr interference. As a result, the lower Ca/Sr ratio in Delniwa reflected a higher translocation of Sr, which may compromise Ca-dependent processes.

Ca plays a central role in cell wall stabilization, plasma membrane integrity, and signal transduction. Sr, when accumulated in excessive amounts, can fill Ca-binding sites in pectin cross-linking and cell wall stabilization, potentially weakening structural integrity. The Sr concentrations measured in raspberry fruits (1.57–16.03 mg/kg) fall within ranges reported for plant-based foods and reflect natural soil–plant transfer processes. Although Sr was not classified as an essential dietary element, its interaction with Ca metabolism has been documented, mainly under elevated exposure [41,42]. The detected levels did not indicate excessive accumulation and were not expected to pose immediate dietary risks. However, the dependence of Sr uptake on the soil geochemical background supports the demand for continued monitoring in the Sr-rich environments [11].

At the physiological level, the observed differences between cultivars suggested that Opal possesses more efficient Ca transport systems and compartmentalization strategies, thereby limiting Sr mobility into fruits. Delniwa, on the other hand, appeared more prone to Sr co-transport, which may be linked to less selective cation channels or differences in vacuolar sequestration mechanisms.

From a nutritional standpoint, Sr is not considered an essential element in human diets, though it can mimic Ca in metabolic pathways [41]. Nevertheless, excessive or chronic exposure could disrupt Ca homeostasis, mainly in populations already at risk of dietary Ca deficiency [42]. The consistently higher Sr levels in Delniwa fruits underlined the need for careful monitoring in regions with Sr-rich soils or where fertilizers may contribute to additional Sr loading.

The interplay between Ca and Sr in raspberry cultivation reflected the dual influence of soil chemistry and genotype. Although soils strongly favoured Ca accumulation, genetic selectivity determined the degree to which Sr was co-transported into fruits. Opal emerged as the cultivar better adapted to maintaining high Ca/Sr ratios in fruits, thereby ensuring firmer texture and reduced risk of Sr interference. On the other hand, Delniwa showed a tendency for higher Sr accumulation, warranting closer attention in biofortification and food safety contexts.

### 2.7. Implications for Raspberry Cultivation, Nutritional Quality, and Food Safety in Romania

The present findings provided an integrated perspective on agronomic management, fruit nutritional value, and food safety considerations in raspberry (*Rubus idaeus* L.) cultivation under Romanian pedoclimatic conditions. The results demonstrated that mineral composition in raspberries was governed by a complex interaction between soil geochemistry, cultivar-specific uptake selectivity, and competitive ion transport processes. These interactions had direct implications for sustainable production systems, cultivar selection strategies, and consumer health.

From an agronomic standpoint, the pronounced differences observed between cultivars highlighted the necessity of site-specific soil assessment and tailored fertilization management. Long-term studies have shown that fertilization regimes can significantly influence soil buffering capacity, nutrient availability, and ion competition dynamics, thereby affecting plant uptake patterns and fruit mineral composition [43,44]. In the present study, the cultivar Opal exhibited higher accumulation of essential macronutrients, primarily Ca, Mg, and K, suggesting a stronger physiological selectivity toward nutritionally beneficial elements. Such characteristics make this cultivar well suited for production systems targeting high fruit quality, improved firmness, and extended postharvest stability. On the other hand, Delniwa exhibited a higher tendency to accumulate non-essential trace elements such as Sr and Li, indicating that its cultivation may require stricter soil monitoring, especially in regions characterized by carbonate-rich soils or elevated geogenic trace element backgrounds.

These findings underline the importance of maintaining balanced soil ionic ratios, particularly Ca/Sr and K/Na, which directly regulate competitive uptake processes due to similarities in ionic radius and transport pathways [25,45]. Adequate fertilization practices that sustain favorable cation balance can therefore mitigate excessive translocation of non-essential elements into edible tissues. Furthermore, cultivar choice emerges as a critical agronomic tool for managing mineral composition in raspberries. Opal ability to preferentially accumulate essential nutrients while limiting Sr uptake suggests its suitability for regions where fruit quality, storage performance, and nutritional safety are prioritized. Conversely, Delniwa may be more appropriate for areas with low soil Sr concentrations but requires careful monitoring in mineral-rich environments. These genotype-dependent differences also provide valuable insights for breeding programs, which increasingly aim to develop cultivars combining high nutrient selectivity with reduced accumulation of potentially harmful trace elements [46].

From a nutritional perspective, raspberries represent a valuable dietary source of essential minerals. The high K content observed in this study confirmed the important contribution of raspberries to cardiovascular health, neuromuscular function, and electrolyte balance, as adequate K intake is associated with reduced hypertension risk [47]. Similarly, Mg concentrations reinforced the role of raspberries in supporting enzymatic activity, energy metabolism, and bone health [47]. Ca, although present at lower concentrations than K and Mg, remained critical both for human nutrition and for fruit structural integrity, contributing to firmness and shelf life.

The detection of Li at trace levels is noteworthy from a nutritional standpoint. Although not considered an essential nutrient, low dietary Li intake has been associated with potential neuroprotective and mood-stabilizing effects, particularly through modulation of neurotransmitter systems and oxidative stress pathways [48]. The slightly higher Li accumulation observed in Delniwa suggested genotype-dependent uptake mechanisms. Nevertheless, the detected concentrations remained within the ranges typically reported for the plant-derived foods and were not associated with any known adverse effects under normal dietary exposure conditions [11,41,42].

At controlled levels, Sr can support bone metabolism and has been used therapeutically in osteoporosis treatment, but excessive dietary exposure may interfere with calcium homeostasis [49]. The higher Sr concentrations observed in Delniwa fruits emphasized the need for continuous monitoring of soil–plant transfer pathways, mainly in the regions where natural geological conditions or anthropogenic inputs increase Sr availability. Na levels in both cultivars remained low, representing a favorable nutritional attribute given the established relationship between excessive sodium intake and cardiovascular disease risk.

Beyond their nutritional importance, raspberries may also serve as effective bioindicators of trace element dynamics in agricultural ecosystems. Their demonstrated capacity to accumulate Li and Sr suggests potential applications in environmental monitoring of soil contamination or geochemical variability [25]. As raspberry cultivation continues to expand in Romania in response to increasing market demand, the adoption of sustainable agronomic practices, including balanced fertilization, precision irrigation, and organic soil amendments, will be essential for maintaining soil quality while minimizing excessive uptake of non-essential elements.

In the broader context of the European market competitiveness, fruit mineral composition represents an important quality parameter influencing consumer acceptance, shelf life, and nutritional value [50]. Cultivars exhibiting higher Ca/Sr and K/Na ratios, such as Opal, are more likely to meet both technological and safety standards, while also providing opportunities for functional food positioning due to their favorable mineral profiles. Overall, the integration of soil monitoring, cultivar selection, and sustainable management practices is essential to ensure both the productivity and safety of raspberry production systems in Romania.

From a practical cultivation perspective, the present investigation highlighted several actions that may be considered by the growers. A balanced soil nutrient availability, mainly for K and Mg may be essential for optimizing fruit nutritional quality, considering their high transfer efficacity to fruits. On the other hand, an increased Ca supply in the soil may not directly enhance the Ca content from the fruits, due to its limited mobility, evidencing that other alternative approaches such as a foliar application or cultivar selection should be considered.

Moreover, the accumulation of non-essential elements (Li and Sr) may be primarily controlled by the soil geochemical background rather than by the fertilization, emphasizing the importance of site selection and soil monitoring. Finally, the cultivar choice may constitute a key management tool, as differences between Opal and Delniwa indicate a variable selectivity toward the essential versus non-essential elements. All these findings support the idea of an integrated management strategy which should combine the soil assessment, targeted fertilization, as well as the cultivar selection to improve the fruit quality while reducing the potential risks.

## 3. Materials and Methods

### 3.1. Study Locations and Sampling Methodology

Raspberry fruits and their corresponding cultivation soils were collected from two distinct agricultural regions in Romania (Bucharest and Vâlcea), over a two-year monitoring period (2024–2025). The fruit samples comprised two commercially relevant raspberry (*Rubus idaeus* L.) cultivars, namely Opal and Delniwa. Opal and Delniwa were cultivated within the experimental plots under both fertilized and unfertilized soil conditions to enable a comparative assessment of mineral uptake dynamics.

Within each agroecosystem, two cultivars (Opal and Delniwa) were systematically monitored. During the 2024 growing season, sampling was conducted under baseline agronomic conditions without the application of experimental fertilizers, thereby providing a reference framework for natural soil–plant transfer processes. In the subsequent 2025 season, two biological fertilizer treatments, F1 and F2, were applied in pre-established experimental plots to evaluate their influence on soil properties and elemental uptake.

F1 is an organic biofertiliser produced via anaerobic digestion and, in this study, it was applied only as the digestate fraction. It provides a readily available pool of nutrients together with a biodegradable organic matrix that can support nutrient supply to plants and stimulate microbial-mediated soil processes. F1 was developed by Prof. Emilia Dunca and her team (Department of Environmental Engineering and Geology, Faculty of Mining, University of Petroșani) within the CeSoH research project, funded by the Research and Innovation Program PNRR-III-C9-2022–I5 (European Union—NextGenerationEU), Grant No. 760005/30.12.2022, Project Code 2: Restoring soil health on unproductive land through biomass crops for sustainable energy—Soil–Biomass–Sustainable [51]. F2 is an organo–mineral biocomposite obtained by integrating mineral/industrial alkaline constituents, including Ca–Mg silicate-bearing fractions, with organic agro-industrial co-products derived from pomace and yeast residues. The formulation was optimised using a statistical mixture/blending strategy to tune key functional attributes, notably pH buffering potential, moderated nutrient release behaviour, and favourable organic functional-group characteristics. F2 was developed by Prof. Gabriela-Elena Bahrim and her team (Faculty of Food Science and Engineering, Dunărea de Jos University of Galați) within the same CeSoH programme (PNRR-III-C9-2022–I5, European Union—NextGenerationEU; Grant No. 760005/30.12.2022), Project Code 4: Innovative and emerging solutions for smart valorisation of residual resources impacting health and safety of the soil–food axis (InnES—Innovation, Emerging, Solutions–Soil). The exact compositional ratios of both formulations are not disclosed due to an ongoing patent application. The experimental design targeted an annual nutrient load of 85–50–65 kg/ha (N–P_2_O_5_–K_2_O) at Vâlcea and 100–25–55 kg/ha at Bucharest. To optimize uptake and align with the phenological stages of the *Rubus idaeus* L. varieties, the total calculated dosage for both F1 and F2 was administered in two synchronized split-applications: the first during the pre-vegetative phase and the second prior to anthesis. The elemental composition of both F1 and F2 was characterized in terms of major nutrients (N, P, K), while the trace elements (Li and Sr) were not quantified; therefore, their potential contribution through fertilization could not be directly assessed.

The investigated matrices were selected due to the recognized nutritional and functional value of raspberries, which are rich in bioactive compounds, including antioxidants (e.g., ellagic acid, anthocyanins, quercetin), polyphenols (flavonoids, tannins, resveratrol), and carotenoid pigments (lutein, zeaxanthin, β-carotene). Concentrations of lithium (Li), strontium (Sr), calcium (Ca), sodium (Na), potassium (K), and magnesium (Mg) were determined in a total of 119 fruit samples collected during the 2024–2025 seasons, together with eight corresponding soil samples collected across three years (2023–2025).

To investigate vertical distribution patterns of trace elements, the soil samples collected in 2023 and 2024 were obtained from two standardized depths (0–20 cm and 20–40 cm). To represent the effective root-zone availability, these measurements were averaged to obtain a depth-integrated soil concentration. For the 2025 sampling campaign, a composite sample representing the entire 0–40 cm soil profile was collected and used directly as the integrated soil concentration. This stratified sampling design enabled evaluation of depth-dependent variations in elemental availability and mobility within the soil profile.

Raspberry fruits were collected at full ripeness. The fruit dataset included 61 Opal and 58 Delniwa samples, originating from two geographically and environmentally contrasting regions: Bucharest (n = 84), representing a highly anthropogenically influenced area, and Vâlcea (n = 35), characterized by comparatively lower pollution levels. Samples were collected across multiple growing periods and under both conventional and fertilized soil management regimes, allowing assessment of the influence of environmental conditions and agronomic practices on mineral and trace element uptake in raspberry fruits. The plantations comprised two distinct production settings: an organic management plot located at INMA, Bucharest (“organic”) and an agroforestry-based system at Vlădești, Vâlcea County (“agroforestry”). Both sites were established in spring 2023 (second decade of March) using the same two cultivars (Opal and Delniwa) and covered approximately 1000 m^2^ per site, providing a controlled framework to compare cultivar responses across contrasting pedoclimatic and land-use conditions.

### 3.2. Sample Preparation

The reagents, including hydrochloric acid (37%), nitric acid (65% for analysis EMSURE) were purchased from Merck KGaA (Darmstadt, Germany). Ultrapure water was produced using an Elga Veolia PURELAB Flex3 system (Elga LabWater, High Wycombe, UK). All chemicals and reagents were of spectroscopic grade; the ICP Multi-Element Standard Solution IV Certipur, with a certified value of 1000.0 ± 20.0 mg·L^−1^ (Merck KGaA Frankfurter, Darmstadt, Germany) was used in quantitative analysis for both trace and mineral elements for the calibration curve. All the investigated calibration curves were characterized by a high correlation coefficient (R^2^ > 0.995).

Due to the complex composition of the samples, strong acid digestion was required to ensure complete metal solubilization and the conversion of solid particles into a homogeneous liquid phase prior to elemental analysis. Soil and raspberry samples were oven-dried at 60 °C for 12 h or until constant mass, ground using a stainless-steel mill, and sieved through a 0.45 mm mesh. Sample digestion was performed according to an optimized internal laboratory protocol: 0.5 g of raspberry sample was digested with 10 mL of 65% HNO_3_, while 0.5 g of soil sample was digested with 10 mL of aqua regia (2.5 mL of 65% HNO_3_ and 7.5 mL of 37% HCl) [52]. The acid digestion of the samples (raspberry and soil) was performed in a microwave oven, model MARS6 CEM One Touch (CEM Corporation, Matthews, NC, USA), with the controlled program (pressure and temperature) for 15 min at 175 °C for soil samples and 15 min at 200 °C. After complete digestion and cooling, the samples were filtered, transferred into 50 mL graduated polypropylene tubes, and diluted to volume with deionized water.

For the soluble cation’s determination, soil suspensions were prepared using 250 mL ultrapure water and 50 g of soil into 500 mL Erlenmeyer flask. The soil/water mixture was then stirred for 15 min on the agitator and then filtered. Exchangeable cations were determined by extracting 10 g of previously dried soil that had been passed through a 2 mm sieve. The soil was transferred into a 250 mL Erlenmeyer flask and 100 mL of ammonium acetate solution was added using a pipette. The suspension was shaken for 15 min, allowed to rest for 24 h, and then filtered on through dry filter paper to obtain the exchangeable cations extract.

### 3.3. Analytical Procedure

Li and Sr identification and quantification were performed using inductively coupled plasma optical emission spectroscopy (ICP-OES; PlasmaQuant 9100, Analytik Jena GmbH, Jena, Germany). The standard sample introduction system was replaced with inert components, including a ceramic D-torch and ceramic injector, along with a PFA cyclonic spray chamber and concentric nebulizer (OpalMist^®^ and DuraMist^®^, Glass Expansion, Melbourne, Australia), to minimize instrumental multielement background. The ICP-OES operating parameters are summarized in the Supplementary Material S1, Table S1.

The most sensitive emission wavelength of the analysed elements, which should work as close as possible to the detection limit, was chosen based on the specialized literature [53,54,55]. Quantification was carried out using external calibration with multi-element standard solutions in the ranges of 0.5–10.0 mg/L for the soil matrix and 0.5–100.0 µg/L for the raspberry matrix. Calibration curves showed acceptable linearity (0.99897–0.99972) within the investigated concentration ranges and was verified using the Mandel test [55,56].

To secure accuracy and reproducibility, the instrumental parameters were carefully adjusted. The plasma gas and argon gas flow rates were configured at 12.0 L/min, while the flows of auxiliary and nebulizer gas were kept at 0.7 L/min. The RF power was adjusted to 1200 W, and a high level was maintained for the purge gas flow rate. A peristaltic pump flow rate of 1.1 mL/min and a wash rate of 1.5 mL/min were applied, with a rinsing time of 60 s to prevent cross-contamination. The read delay was set to 30 s, and the integration time was 3 s per replicate. All measurements were performed in triplicate. Elemental concentrations in raspberry samples are reported on a dry weight (DW) basis.

The quality assurance/quality control QA/QC of the analytical method was validated using certified reference materials and spike recoveries, confirming high accuracy (Recovery % from 96.228% to 100.639%) and precision (% RSDr typically below 5%). Detailed analytical performance parameters, including R^2^ values, limits of detection (LOD), limits of quantification (LOQ), and strategies for spectral interference correction (where necessary), are presented for both soil and raspberry matrices in Supplementary Material S1, Table S2.

Ca, Mg, Na and K identification and quantification was carried out using flame atomic absorption spectroscopy (AAS). Atomic Absorption Spectrophotometer (AAS) NOVAA 300 model (Analytik Jena GmbH, Jena, Germany) with Air-C_2_H_2_ flame type of an average fuel flow rate of between 0.8–4.0 L/min and the support gas flow rate between 13.5–17.5 L/min was used for sample analysis. Hallow cathode lamps of the different metals were used as the radiation sources and the analytical measurements based on time-averaged absorbance. Resonance lines at 422.7, 285.2, 589.0 and 766.5 nm were employed for Ca, Mg, Na and K. Lamp intensity of 4–6 mA and band pass of 0.2–0.5 nm were used according to the equipment manufacturer’s recommendations. Prior to each series of measurements, a calibration line was created for each element.

### 3.4. Transfer Factors Calculation

Transfer factors (TFs) were used to assess element bioavailability and plant uptake efficiency and they were defined as the ratio between element concentrations in fruits and soils.

(1) $$TF=\frac{C_{fruit}}{C_{soil}}$$

where C_fruit_ was the metal concentration in fruit (raspberry) and C_soil_ was the metal concentration in soil.

TFs for Li and Sr were calculated using total soil concentrations, whereas exchangeable soil fractions were considered for Ca, Mg, Na, and K, to reflect the plant-available pools of these macronutrients.

### 3.5. Statistical Analysis and Data Visualization

The applied methodological framework was designed to ensure a coherent and internally consistent evaluation of multiyear interactions between soil metal availability and the subsequent accumulation of these elements in raspberry fruits. This approach integrated soil and plant matrices collected at different temporal stages within a unified analytical structure based on a clearly defined chronological dependency. Rather than being interpreted as independent datasets, soil samples were treated as components of a continuous temporal sequence, whereby the chemical status of the soil recorded in year t–1 was considered the baseline substrate influencing the physiological and biochemical conditions governing fruit development in year t.

Given the non-normal distribution, heteroscedasticity, and multifactorial structure of the data (site × year × fertilization × variety), emphasis was placed on non-parametric effect size estimation and distributional analysis rather than multiple pairwise comparisons. In this respect, boxplots were further interpreted in terms of variability, central tendency, and group separation, without the use of significance lettering.

Accordingly, soil data obtained in 2023 were regarded as the agronomic and geochemical reference for the raspberry crop harvested in 2024, while soil data from 2024 provided the corresponding baseline for fruit production in 2025. The soil dataset collected in 2025 was not used in transfer calculations; instead, it was evaluated exclusively as post-harvest residual soil to assess changes in the metal balance following two consecutive cultivation cycles.

The temporal and stratified sampling design enabled a systematic assessment of depth-dependent metal distribution patterns and their evolution over successive cultivation years, while maintaining a consistent chronological linkage between soil conditions and fruit mineral composition.

Given the chemical heterogeneity of the soil datasets, which included soluble, exchangeable, and directly measured elemental fractions, the study applied the widely accepted assumption in environmental geochemistry and plant nutrition research that the soluble fractions of Ca, Mg, and Na, the exchangeable fraction of K and the directly measured available concentrations of Li and Sr constitute the biologically accessible pool of each metal for root uptake. To obtain a physiologically meaningful and vertically integrated descriptor of the soil metal availability, the measurements at 0–20 cm and 20–40 cm were arithmetically averaged whenever both depths were available, yielding:

(2) $$C_{soil,mean}\left(i,t,m\right)=\frac{C_{soil,bio}\left(i,t,m,0-20\right)+C_{soil,bio}\left(i,t,m,20-40\right)}{2}$$

where letter i denotes the cultivation site, t the soil sampling year, and m the metal species; the bioavailable metal concentration measured in depth interval d was represented as C_soil,bio_ (i,t,m,d).

In the specific case of the 2025 fertilized sample, this integrated concentration was accepted without further transformation. This vertically averaged concentration was taken to represent the effective soil metal availability experienced by plant roots during the early vegetative and reproductive growth stages.

Raspberry samples harvested in 2024 and 2025 were analysed for the same suite of metals, with results expressed uniformly in mg/kg. The corresponding fruit metal concentration was denoted $C_{fruit}(i,t,v,m)$ where i—the cultivation site, t—the fruit sampling year, v—raspberry variety, m—the specific metal species under investigation.

The deterministic temporal linkage between soil and fruit was formalized through the definition of a baseline soil concentration:

(3) $$C_{soil,base}\left(i,t,m\right)=C_{soil,mean}(i,t-1,m)$$

which reflects the agronomic principle that the chemical conditions of the soil preceding the growing season govern metal mobilization, root uptake, vascular transport, and eventual translocation into raspberry fruit tissues.

Within this conceptual framework, the transfer factor (TF), a dimensionless index widely used in environmental chemistry and risk assessment to quantify soil-to-plant transfer efficiency, was defined as:

(4) $$TF\left(i,t,v,m\right)=\frac{C_{fruit}\left(i,t,m,variety\right)}{C_{soil,base}\left(i,t,m\right)}$$

where C_fruit_ was the metal concentration in fruit (raspberry) and C_soil_ was the metal concentration in soil.

It provides a measure of the degree to which the metal m becomes enriched in raspberries relative to its bioavailable concentration in the corresponding baseline soil. Values markedly greater than unity indicated enhanced accumulation capacity, values near unity suggested quasi-equilibrium between soil availability and fruit concentration, and values substantially below unity pointed to limited translocation or restricted root uptake.

The raw soil and raspberry fruit datasets originated from separate spreadsheets with distinct structural conventions; therefore, they were subjected to an extensive data harmonization and restructuring procedure aimed at eliminating inconsistencies in formatting, nomenclature, and variable representation. Soil data were first standardized through whitespace removal, depth normalization, and extraction of the bioavailable metal fractions, after which all measurements were converted into a long-format relational table containing one observation per location, year, depth, metal, and bioavailable concentration.

Fruit data underwent an analogous transformation, in which metal-specific analytical columns were consolidated into a unified long-format dataset containing one observation per fruit sample and metal. These normalized datasets were subsequently aligned via a relational join based on location, year, and metal, ensuring, through the constraint t_fruit_ = t_soil_ + 1, that each raspberry fruit concentration could be associated with the correct baseline soil value. In instances where no baseline soil measurement existed (for example, sites lacking a soil sampling in the year preceding fruit harvesting), the observations were flagged as lacking baseline soil information, thus remaining suitable for fruit-only analyses such as variability assessment, inter-metal correlation studies, or comparisons across varieties, while being excluded from analyses requiring explicit soil–plant transfer metrics.

Through these sequential procedures of normalization, vertical integration, temporal alignment and relational merging, the study produced a unified and internally coherent master dataset in which all raw and depth-averaged soil measurements were systematically combined with the corresponding raspberry fruit metal concentrations, the computed baseline soil values, the resulting transfer factors and the metadata describing sampling completeness and soil–fruit linkage status.

Descriptive statistics were calculated to summarize the distribution of both raspberry fruit metal concentrations and transfer factors across different combinations of metal, location, raspberry variety, and sampling year.

For each combination of metal, location, variety and sampling year, the set of observations was denoted $x_{g}={\left\{x_{g,1}\right.,x}_{g,2},x_{g,3},\ldots ,\left.x_{g,n_{g}}\right\}$, indexes the grouping level (e.g., metal, metal x location, metal x variety x year), where n_g_ represents the number of observations in group g, and x_g,j_ represents the measured value (either fruit metal concentration or TF for the jth sample in the group.

The arithmetic mean was calculated as the sum of all observations divided by the number of samples in that group:

(5) $$\bar{x_{g}}=\frac{\sum_{j=1}^{n_{g}}x_{g,j}}{n_{g}}$$

where x_g,j_—measured value for the jth sample in the group and n_g_—group size.

After ordering the observation $x_{g,1}\leq x_{g,2}\leq \cdots \leq x_{g,n_{g}}$, the median $\tilde{x_{g}}$ was given by:

(6) $$\tilde{x_{g}}=x_{g,\frac{n_{g}+1}{2}},if\;n_{g}=2k+1$$

and

(7) $$\tilde{x_{g}}=\frac{x_{g,\frac{ng}{2}}+x_{g,\frac{ng}{2}+1}}{2},if\;n_{g}=2k$$

The within-group variability was quantified using the unbiased sample standard deviation ($s_{g})$:

(8) $$s_{g}=\sqrt{\frac{1}{n_{g}-1}\sum_{j=1}^{n_{g}}{{(x}_{g,j}-\bar{x_{g}})}^{2}}$$

where $\bar{x_{g}}$—mean of group g.

The first and third quartiles, $Q_{1,g}$ and $Q_{3,g}$, were defined as 25th and 75th percentiles of empirical distribution of $\left\{x_{g,j}\right.\}$, respectively. The interquartile range (IQR) was then calculated as:

(9) $${IQR}_{g}=Q_{3,g}-Q_{1,g}$$

providing a robust measure of dispersion that is less sensitive to extreme values than the standard deviation.

To express relative variability independent of the absolute magnitude of the mean, the coefficient of variation (CV) was computed for each group:

(10) $${CV}_{g}=\frac{s_{g}}{\bar{x_{g}}}$$

where s_g_—standard deviation of group g and $\bar{x_{g}}$—mean of group g.

Higher CV values indicate higher relative dispersion around the mean.

Descriptive statistics were obtained for each metal, as well as for metal-by-location, metal-by-variety, metal-by-year, and selected three-way combinations (metal × location × year, metal × variety × year). For TF, descriptive statistics were computed only for those groups in which a valid baseline soil concentration was available, whereas fruit metal concentration statistics were calculated for the complete dataset.

For the Vâlcea site, soil measurements for the 2024 growing season were not available. Consequently, the most recent pre-cultivation soil dataset (2023) was used as the primary reference for the calculation of transfer (TF), soil background (SBI) and dilution (DI) indices.

In addition, a supplementary trend-based interpolation was explored as part of a sensitivity analysis. This approach assumed that relative interannual changes observed at the Bucharest site reflected broader regional forcing and could be proportionally transferred to the Vâlcea site. Estimated 2024 soil concentrations for Vâlcea were thus obtained by scaling the 2023 Vâlcea values using the element-specific 2024/2023 soil concentration ratios derived from the Bucharest site. Interpolated values were used exclusively for sensitivity testing and did not replace measured soil data.

To assess whether metal concentrations in raspberries and the corresponding transfer factors exhibited statistically significant differences across cultivation sites, raspberry varieties, sampling years and metals, inferential statistics were applied to the grouped datasets generated in the descriptive step. Because several groupings involved more than two levels (e.g., multiple varieties, multiple locations), and because the underlying distributions showed moderate to strong deviations from normality, two complementary frameworks were used: parametric one-way analysis of variance (ANOVA) when assumptions were approximately satisfied, and the non-parametric Kruskal–Wallis test when normality or homoscedasticity was not met.

Assuming that $N=\sum_{g=1}^{k}n_{g}$ is the total sample size, ANOVA decomposes the total variability into between-group and within-group components:

(11) $${SS}_{between}=\sum_{g=1}^{k}n_{g}{\left(\bar{x_{g}}-\bar{x}\right)}^{2}$$

where $\bar{x_{g}}$—the group mean; and $\bar{x}$—the overall mean.

(12) $${SS}_{within}=\sum_{g=1}^{k}\sum_{j}^{n_{g}}{\left(x_{g,j}-\bar{x_{g}}\right)}^{2}$$

F-statistic was defined as:

(13) $$F=\frac{\frac{{SS}_{between}}{k-1}}{\frac{{SS}_{within}}{N-k}}$$

To examine the degree of the linear and monotonic association among metal concentrations in raspberry fruits and, where applicable, their corresponding transfer factors, correlation coefficients were computed for each metal pair within the set of variables under analysis. Let X and Y denote two random variables representing the distributions of metal concentrations (or TF values) across all observations in the dataset.

The Pearson product–moment correlation coefficient was computed as:

(14) $$r_{XY}=\frac{\sum_{j=1}^{n}\left(x_{j}-\bar{x})(y_{j}-\bar{y}\right)}{\sqrt{\sum_{j=1}^{n}{\left(x_{j}-\bar{x}\right)}^{2}}\sqrt{\sum_{j=1}^{n}{\left(y_{j}-\bar{y}\right)}^{2}}}$$

where $x_{j}$, $y_{j}$—paired observations of variables X and Y and $\bar{x},\bar{y}$—sample means.

Pearson’s r measures linear association and takes values in the range $\left[-1,1\right]$.

Because several variables exhibited non-normal distributions and highly skewed tails (particularly Li, Sr, and TF values), Spearman’s rank correlation coefficient was additionally computed:

(15) $$\rho_{XY}=\frac{\sum_{j=1}^{n}\left(R_{x_{j}}-\bar{R_{x}})(R_{y_{j}}-\bar{R_{y}}\right)}{\sqrt{\sum_{j=1}^{n}{\left(R_{x_{j}}-\bar{R_{x}}\right)}^{2}}\sqrt{\sum_{j=1}^{n}{\left(R_{y_{j}}-\bar{R_{y}}\right)}^{2}}}$$

where $R_{x_{j}},R_{y_{j}}$—ranks of the observations.

Spearman’s $\rho_{XY}$ quantifies monotonic, not necessarily linear, associations.

The first stage of preprocessing involved consolidating all relevant observations into a coherent analytical framework. Each record was defined by a unique combination of sampling locality, raspberry variety, fertilization treatment, and sampling year. Variables corresponding to metallic elements (e.g., Ca, K, Li, Mg, Na, and Sr) were automatically identified through string matching of their concentration units (“mg/kg”). The cleaning process focused on harmonizing column names, converting numerical data from text formats, and removing records with missing or incomplete entries to preserve data integrity and ensure compatibility with statistical routines.

To ensure comparability among analytical variables, all numeric fields were converted into standardized floating-point format using consistent English decimal notation. Non-numeric or malformed entries were not detected during systematic validation, and missing values were handled according to predefined inclusion criteria. The resulting dataset was thus numerically coherent, internally consistent, and suitable for subsequent statistical and visualization analyses.

To ensure a robust and reproducible evaluation of metal concentration variability in raspberry fruits, all statistical analyses and graphical representations were performed using the Python programming environment (Python 3.12.3, Python Software Foundation, Wilmington, DE, USA), relying primarily on the Pandas 2.3.3 library (WatchGuard Technologies, Seattle, WA, USA), SciPi (Open-source scientific computing library for Python, Version 1.13.1; NumFOCUS, Austin, TX, USA) for data manipulation and aggregation and Plotly 6.3.1 (Plotly Technologies Inc., Montreal, QC, Canada) for high-resolution, interactive visualization.

Artificial intelligence (AI)-assisted tools were used in a limited capacity to support language refinement and improve clarity of expression. These tools were not used for data analysis, data interpretation, or generation of scientific content. All results, interpretations, and conclusions presented in this study were developed exclusively by the authors.

## 4. Conclusions

This study provided a comprehensive multielement assessment of soil–fruit transfer processes in raspberry (*Rubus idaeus* L.), integrating essential macroelements (Ca, Mg, Na, and K) and trace elements (Li and Sr) within a multiyear field framework. The results demonstrated that the element accumulation in raspberries followed two fundamentally distinct regimes shaped by physiological demand and geochemical constraints.

The results highlighted a clear and consistent hierarchy of the element transfer to fruits, expressed as K ≫ Mg > Na ≈ Sr > Ca ≫ Li, indicating the combined influence of the physiological demand, element mobility, and transport constraints. Spatial and temporal variability emerged as the dominant control on the metal transfer dynamics. The site-specific soil geochemistry strongly influenced the trace element availability, while the interannual changes in dilution intensity significantly modulated transfer efficacity, mainly for Li and Na. The transfer factors proved to be more sensitive indicators for the environmental variability than for the absolute fruit concentrations, highlighting their suitability for assessing soil–plant transfer.

The varietal differences were detectable, but secondary to environmental drivers. The cultivar Opal demonstrated higher selectivity for the essential nutrients and maintained more favourable Ca/Sr ratios, indicating higher physiological discrimination against Sr. In comparison, Delniwa exhibited a higher susceptibility to the trace element co-transport, underscoring the role of genotype in modulating element selectivity.

From the agronomic and nutritional perspective, the determined concentrations of Li and Sr remained well below toxicological thresholds, indicating no immediate food safety concerns. Nevertheless, their presence confirmed the potential of raspberries to serve as bioindicators of soil trace element dynamics.

From the food safety perspective, the absence of an excessive accumulation of Li and Sr n fruits evidenced a low immediate risk under the studied conditions; nevertheless, their dependence on the soil background underscored the demand for continued monitoring, mainly in the regions with increased geogenic concentrations.

Future research should focus on the mechanistic basis of the element-specific uptake and translocation, also considering the soil–plant interactions, root transfer systems, and environmental variability. Expanding the multiyear and multisite datasets, as well as integrating additional trace elements and different berry species, would create a more comprehensive understanding of the mineral dynamics in the fruit crops and support the development of a sustainable and safe agricultural practices.

## Figures and Tables

**Table 1:** Total (Li, Sr) and exchangeable (Ca, Mg, Na, K) element concentrations in soils from the investigated regions (mean ± SD).

Region	Year	Li (mg/kg)	Sr (mg/kg)	Exchangeable Ca (mg/kg)	Exchangeable Mg (mg/kg)	Exchangeable Na (mg/kg)	Exchangeable K (mg/kg)
Bucharest	2024	4.10 ± 0.00	13.37 ± 0.00	5233.00 ± 0.00	404.20 ± 0.00	179.35 ± 0.00	258.55 ± 0.00
Bucharest	2025	2.63 ± 0.00	7.25 ± 0.00	4438.87 ± 0.00	273.95 ± 0.00	59.83 ± 0.00	190.42 ± 0.00
Bucharest (overall)	2024–2025	3.08 ± 0.68	9.15 ± 2.84	4684.67 ± 369.33	314.27 ± 60.58	96.82 ± 55.59	211.51 ± 31.69
Vâlcea	2025	1.67 ± 0.00	13.78 ± 0.00	2084.64 ± 0.00	624.69 ± 0.00	200.88 ± 0.00	358.92 ± 0.00

Li and Sr represent total soil concentrations, while Ca, Mg, Na, and K correspond to exchangeable fractions; values are expressed as mean ± standard deviation (SD); “zero” SD values indicate that a single composite soil sample was analysed for the corresponding site and year; Bucharest (overall) represents pooled data from 2024 and 2025.

**Table 2:** Elemental composition of raspberry fruits as affected by variety, region, and fertilization regime (mean ± SD).

Variety	Region	Fertilization	Li (mg/kg)	Sr (mg/kg)	Ca (mg/kg)	Mg (mg/kg)	Na (mg/kg)	K (mg/kg)
DELNIWA	Bucharest	No	0.12 ± 0.15	3.33 ± 1.63	132.72 ± 80.90	191.49 ± 108.65	36.09 ± 28.30	2970.38 ± 3577.16
DELNIWA	Bucharest	Yes	0.54 ± 1.43	2.99 ± 1.11	161.40 ± 74.25	233.71 ± 115.57	21.21 ± 10.31	1447.14 ± 982.40
DELNIWA	Vâlcea	No	0.07 ± 0.19	6.42 ± 1.49	151.68 ± 61.64	227.35 ± 70.11	14.86 ± 5.43	1251.36 ± 730.50
DELNIWA	Vâlcea	Yes	0.00 ± 0.00	3.11 ± 2.60	277.99 ± 87.51	287.76 ± 73.99	22.67 ± 3.60	1022.09 ± 938.76
OPAL	Bucharest	No	0.13 ± 0.27	3.56 ± 2.02	120.37 ± 61.89	198.29 ± 101.23	36.49 ± 31.94	2503.75 ± 2766.24
OPAL	Bucharest	Yes	0.07 ± 0.14	3.49 ± 1.55	142.56 ± 92.24	254.60 ± 85.69	22.06 ± 10.37	1393.09 ± 558.14
OPAL	Vâlcea	No	0.07 ± 0.25	2.94 ± 3.69	157.02 ± 91.29	159.87 ± 102.97	15.28 ± 7.90	1385.37 ± 832.34
OPAL	Vâlcea	Yes	0.03 ± 0.04	8.00 ± 1.09	124.47 ± 175.75	297.50 ± 74.43	22.84 ± 5.93	2238.73 ± 752.58

Values were expressed as mean ± standard deviation (SD) of biological replicates; “zero” SD values indicated single composite fruit samples.

**Table 3:** Soil–fruit transfer factors (TFs) for Li, Sr, Ca, Mg, Na, and K in raspberries as affected by variety, region, and fertilization (mean ± SD).

Variety	Region	Fertilization	TF_Li_	TF_Sr_	TF_Ca_	TF_Mg_	TF_Na_	TF_K_
DELNIWA	Bucharest	No	0.03 ± 0.05	0.35 ± 0.19	0.027 ± 0.018	0.57 ± 0.36	0.30 ± 0.18	12.27 ± 13.59
DELNIWA	Bucharest	Yes	0.20 ± 0.55	0.41 ± 0.15	0.036 ± 0.017	0.85 ± 0.42	0.35 ± 0.17	7.60 ± 5.16
DELNIWA	Vâlcea	No	0.04 ± 0.11	0.47 ± 0.11	0.073 ± 0.030	0.36 ± 0.11	0.074 ± 0.027	3.49 ± 2.04
DELNIWA	Vâlcea	Yes	0.001 ± 0.002	0.23 ± 0.19	0.133 ± 0.042	0.46 ± 0.12	0.113 ± 0.018	2.85 ± 2.62
OPAL	Bucharest	No	0.03 ± 0.07	0.39 ± 0.24	0.025 ± 0.013	0.60 ± 0.34	0.28 ± 0.19	10.71 ± 10.52
OPAL	Bucharest	Yes	0.03 ± 0.05	0.48 ± 0.21	0.032 ± 0.021	0.93 ± 0.31	0.37 ± 0.17	7.32 ± 2.93
OPAL	Vâlcea	No	0.04 ± 0.15	0.21 ± 0.27	0.075 ± 0.044	0.26 ± 0.16	0.076 ± 0.039	3.86 ± 2.32
OPAL	Vâlcea	Yes	0.016 ± 0.027	0.58 ± 0.08	0.060 ± 0.084	0.48 ± 0.12	0.114 ± 0.030	6.24 ± 2.10

Values were expressed as mean ± standard deviation (SD) of biological replicates.

## Data Availability

The original contributions presented in this study are included in the article/Supplementary Materials. Further inquiries can be directed to the corresponding authors.

## Supplementary Materials

The following supporting information can be downloaded at: https://www.mdpi.com/article/10.3390/plants15071107/s1, Supplementary Material S1: Table S1. ICP-OES instrument parameters for multielement determination; Table S2. ICP-OES analytical performance parameters; Supplementary Material S2: Soil-metal concentration; Raspberry-metal concentration.
