# Supplementary material for: Multiyear Soil–Fruit Transfer Dynamics of Macro- and Trace Elements in Raspberry (Rubus idaeus L.) Under Field Conditions
Source: Plants (Basel). 2026 Apr 3;15(7):1107. doi: 10.3390/plants15071107 (PMC13074837; doi:10.3390/plants15071107)
Supplement: Supplementary file 1 [file plants-15-01107-s001.zip › Supplementary Material_1.pdf]

- *Supplementary Material_1* –

Multielement Soil–Fruit Transfer Dynamics in Raspberry (*Rubus idaeus L.)*: Multiyear Evidence on Macro- and Trace Elements Bioaccumulation

Ramona Zgavarogea [1], Nadia Paun [1,*], Claudia Sandru[1], Violeta-Carolina Niculescu [1,*], Ana Maria Nasture [1], Augustina Mirabela Pruteanu [2], Irina-Aura Istrate [3], Oana-Romina Botoran [1]

[1] National Research and Development Institute for Cryogenic and Isotopic Technologies—ICSI Ramnicu Vâlcea, 4th Uzinei Street, P.O. Box Raureni 7, 240050 Ramnicu Vâlcea, Romania; ramona.zgavarogea@icsi.ro (R.Z.); ana.nasture@icsi.ro (A.M.N.), claudia.sandru@icsi.ro (C.S.); oana.dinca@icsi.ro (O.-R.B.)

[2] National Institute of Research—Development for Machines and Installations Designed for Agriculture and Food Industry—INMA, 013813 Bucharest, Romania; pruteanu@inma.ro, pruteanu_augustina@yahoo.com (A.M.P.)

[3] Faculty of Biotechnical Systems Engineering, National University of Science and Technology Politehnica Bucharest, Splaiul Independentei 313, 060042 Bucharest, Romania; irina_aura.istrate@upb.ro (I.-A.I.)

* Correspondence: violeta.niculescu@icsi.ro (V.-C.N.); nadia.pauns@icsi.ro (N.P.)

Table S1. ICP-OES instrument parameters for multielement determination

Parameters/Element	Wavelength (nm)	Gas type	Plasma view	Calibration range, soil matrix	Calibration range, vegetables matrix
Li	670.791	Ar	Radial	0.5 – 10.0 mg/L	0.5–100.0 µg/L
**Sr**	407.771	Ar	Radial		

Table S2. ICP-OES analytical performance parameters

Matrix	Element	R^2	Standards	Matrix Match	LOD (mg/L)	LOQ (mg/L)	Precision (%RSDr)	Uncertainly (%)	Recovery (%)	Spectral Interference Correction
Soil	Li	0.99897	5	Yes	0.00742	0.0225	3.9859	9	100.639	Corrected by spectral separation and background correction
	Sr	0.99968	5	Yes	0.00327	0.0098	1.3328	10	98.518	No correction required
Raspberry	Li	0.99969	5	Yes	0.00304	0. 0912	1.2074	8	96.228	No correction required
	Sr	0.99972	5	Yes	0.01200	0.0360	1.4305	13	97.458	No correction required
